# Development of a promising microbial platform for the production of dicarboxylic acids from biorenewable resources

**DOI:** 10.1186/s13068-018-1310-x

**Published:** 2018-11-09

**Authors:** Heeseok Lee, Changpyo Han, Hyeok-Won Lee, Gyuyeon Park, Wooyoung Jeon, Jungoh Ahn, Hongweon Lee

**Affiliations:** 1 Biotechnology Process Engineering Center, Korean Research Institute of Bioscience and Biotechnology (KRIBB), 30 Yeongudanji-ro, Cheongwon-gu, Cheongju-si, Chungcheongbuk-do 28116 Republic of Korea; 2 Department of Bioprocess Engineering, KRIBB School of Biotechnology, Korea University of Science and Technology (UST), 217 Gajeong-ro, Yuseong-gu, Daejeon, 34113 Republic of Korea

**Keywords:** *Wickerhamiella sorbophila*, Alkane-assimilating yeast, Microbial platform, Dicarboxylic acid, Dodecanedioic acid, Vegetable oil, Genetic engineering tool

## Abstract

**Background:**

As a sustainable industrial process, the production of dicarboxylic acids (DCAs), used as precursors of polyamides, polyesters, perfumes, plasticizers, lubricants, and adhesives, from vegetable oil has continuously garnered interest. Although the yeast *Candida tropicalis* has been used as a host for DCA production, additional strains are continually investigated to meet productivity thresholds and industrial needs. In this regard, the yeast *Wickerhamiella sorbophila*, a potential candidate strain, has been screened. However, the lack of genetic and physiological information for this uncommon strain is an obstacle that merits further research. To overcome this limitation, we attempted to develop a method to facilitate genetic recombination in this strain and produce high amounts of DCAs from methyl laurate using engineered *W. sorbophila*.

**Results:**

In the current study, we first developed efficient genetic engineering tools for the industrial application of *W. sorbophila*. To increase homologous recombination (HR) efficiency during transformation, the cell cycle of the yeast was synchronized to the S/G2 phase using hydroxyurea. The HR efficiency at *POX1* and *POX2* loci increased from 56.3% and 41.7%, respectively, to 97.9% in both cases. The original HR efficiency at *URA3* and *ADE2* loci was nearly 0% during the early stationary and logarithmic phases of growth, and increased to 4.8% and 25.6%, respectively. We used the developed tools to construct *W. sorbophila* UHP4, in which β-oxidation was completely blocked. The strain produced 92.5 g/l of dodecanedioic acid (DDDA) from methyl laurate over 126 h in 5-l fed-batch fermentation, with a productivity of 0.83 g/l/h.

**Conclusions:**

*Wickerhamiella sorbophila* UHP4 produced more DDDA methyl laurate than *C. tropicalis*. Hence, we demonstrated that *W. sorbophila* is a powerful microbial platform for vegetable oil-based DCA production. In addition, by using the developed genetic engineering tools, this emerging yeast could be used for the production of a variety of fatty acid derivatives, such as fatty alcohols, fatty aldehydes, and ω-hydroxy fatty acids.

**Electronic supplementary material:**

The online version of this article (10.1186/s13068-018-1310-x) contains supplementary material, which is available to authorized users.

## Background

Industrial biotechnology, mainly known as white biotechnology in Europe, is a promising solution to replace the production of petroleum-based products that are widely used in daily life. Among various applications, the biotransformation of dicarboxylic acids (DCAs) from renewable resources has attracted much attention. DCAs are high value-added chemicals used as raw materials for a variety of products, such as polyamides (PAs), polyesters, perfumes, plasticizers, lubricants, and adhesives [1]. In particular, medium-chain DCAs (chain length ≥ 10), such as sebacic acid (C_10_) and dodecanedioic acid (C_12_, DDDA), serve as precursors for PA 610 (nylon 6, 10) and PA 612 (nylon 6, 12) synthesis.

**Fig. 1:**
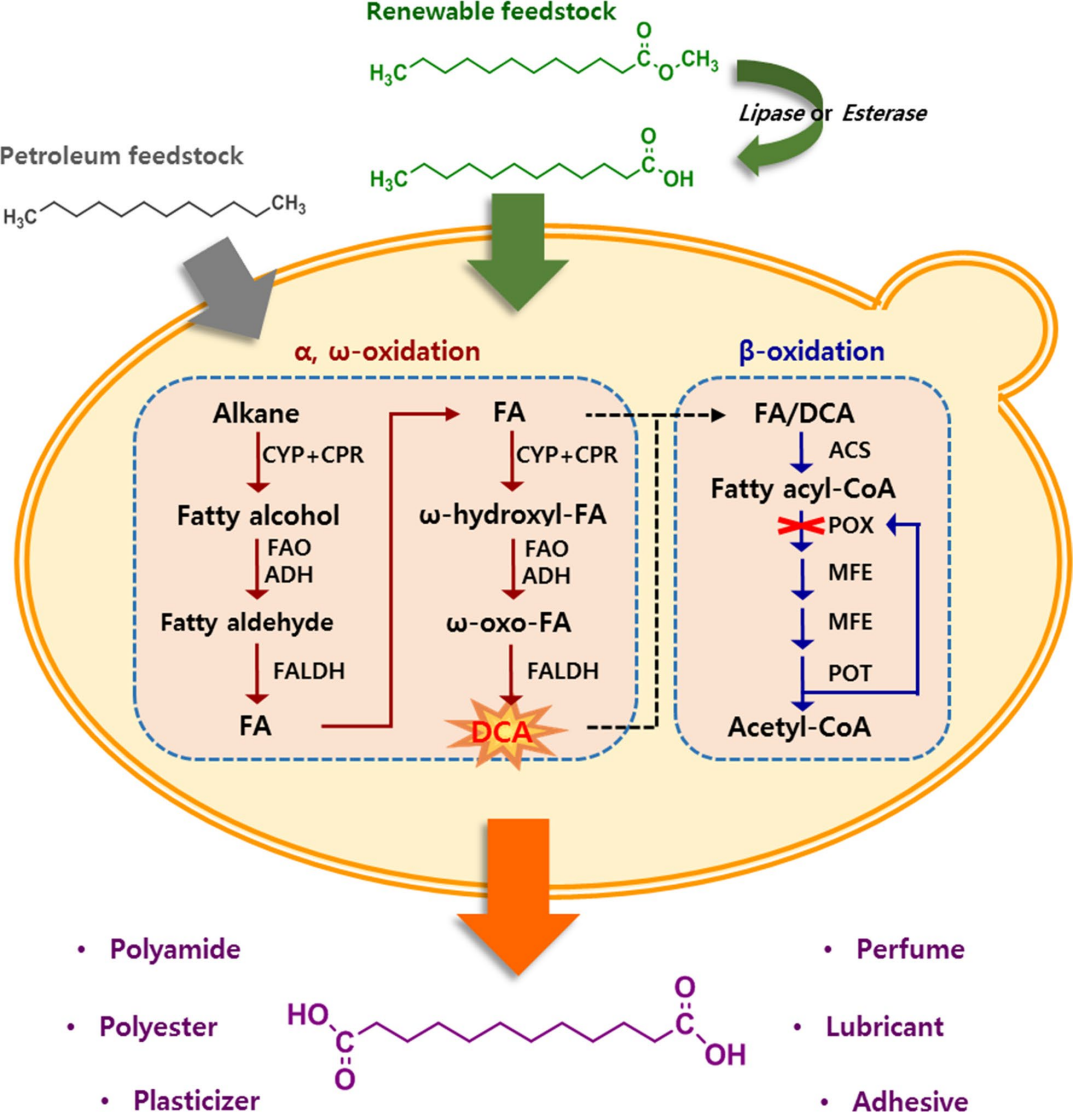
The proposed biological pathway for dicarboxylic acid (DCA) production in *Wickerhamiella sorbophila*. Alkanes are first oxidized to the corresponding fatty alcohol by a cytochrome P450 monooxygenase/cytochrome P450 reductase complex (CYP + CPR). The fatty alcohol is converted into a corresponding fatty aldehyde by fatty alcohol oxidase (FAO) or alcohol dehydrogenase (ADH). Then, the fatty aldehyde is oxidized to fatty acid (FA) by fatty aldehyde dehydrogenase (FALDH). FA methylester is hydrolyzed by extracellular lipase or esterase before primary oxidation, and its corresponding FA is converted to DCA via the same mechanism as alkane. Converted FA and DCA are finally degraded to acetyl-CoA by the successive reactions of acyl-CoA synthetase (ACS), acyl-CoA oxidase (POX), 2-enoyl-CoA hydratase (MFE), 3-hydroxyacyl-CoA dehydrogenase (MFE), and 3-ketoacyl-CoA thiolase (POT). To prevent the continuous catabolism of carboxylic acids to acetyl-CoA, β-oxidation-related genes, POX, are removed

Alkane-assimilating yeasts are hosts for DCA biotransformation. *Candida tropicalis* with a strong ω-oxidizing ability is a representative of this group of yeasts. These microorganisms convert terminal methyl groups of alkanes and fatty acids into carboxylic groups via ω-oxidation and metabolize the corresponding fatty acids and DCAs via the β-oxidation pathway (Fig. 1). Several studies have focused on increasing DCA yield and productivity, for example, by blocking β-oxidation [2, 3] and enhancing ω-oxidation [4, 5] in *C. tropicalis*. This led to the industrialization of the DCA bioprocess using paraffin (alkane) [6]. However, considering the limited availability of petroleum-based resources, such as alkanes, sustainable production of DCA from vegetable oil-based resources is necessary. Among these, methyl laurate (C_12_) is an attractive sustainable resource. It is obtained from the esterification of coconut and palm kernel oil—rich in lauric acid (C_12_)—and is easily accessible for bioprocessing, since it is present in liquid form. Furthermore, medium-chain fatty acid methyl esters, including methyl laurate, are treated as by-products in the biodiesel industry, which relies on various vegetable oils to lower the cetane number associated with the ignition quality of fuel [7]. Consequently, DCA bioprocessing with vegetable oil-based feedstock as a starting material has been currently developed [8]. Unlike alkanes, however, biotransformation using vegetable oil-derived resources poses several difficulties. Cellular toxicity is one of the major obstacles that decrease the overall productivity of the bioprocess. In particular, medium-chain fatty acids (chain length ≤ 12) are highly toxic compounds, which cause cell membrane stress, disruption of the electron transport system, acidification of the cytoplasm, and production of reactive oxygen species [9, 10]. Medium-chain fatty acid methyl esters, such as methyl decanoate and methyl laurate, are hydrolyzed by extracellular lipases or esterases during the conversion phase and the resulting fatty acids cause cellular toxicity [11]. Because of its toxicity, various concentrations of DCA are produced, depending on the carbon chain length of fatty acids or that of methyl esters (Table 1).

**Table 1:** Studies investigating the biotransformation of medium-chain DCAs from alkanes, fatty acids, and fatty acid methyl esters

Strain	Substrate	Analytical method	DCA (g/l)	References
Petroleum-based resources				
*C. tropicalis* ATCC 20962	Dodecane (C_12_)	GC	140^a^	[2]
*C. viswanathii* ipe-1	Dodecane (C_12_)	pH titration	129.7	[51]
*C. tropicalis* CZ-15	Tridecane (C_13_)	pH titration	98	[3]
*C. tropicalis* CGMCC 356	Tridecane (C_13_)	pH titration	153	[34]
*Y. lipolytica* H222ΔPoF	Dodecane (C_12_)	GC	11	[52]
*Y. lipolytica* MTLY 37	Dodecane (C_12_)	GC	8	[53]
*Y. lipolytica* MTLY 37	Tetradecane (C_14_)	GC	2	[53]
Vegetable oil-based resources				
*C. tropicalis* mutant^b^	Methyl decanoate (C_10_)	GC	34.5	[11]
*C. tropicalis* ATCC 20962	Methyl laurate (C_12_)	GC	66	[35]
*C. tropicalis* ATCC 20962	Methyl myristate (C_14_)	GC	210^a^	[2]
*C. cloacae* FERM-P736	Lauric acid (C_12_)	GC	5	[54]
*W. sorbophila* UHP4	Methyl laurate (C_12_)	GC	92.5	This work

^a^Concentration calculated based on the initial volume

^b^Strain tolerant to decanoic acid derived from *C. tropicalis* ATCC 20962

We previously isolated a novel alkane-assimilating yeast *Wickerhamiella sorbophila* from wastewater of a petrochemical factory and attempted to use it to produce DDDA using methyl laurate [12]. The growth of *W. sorbophila* strain capable of ω-oxidation in the presence of mixed fatty acid methyl esters (C_10_–C_16_) was more stable than that of *C. tropicalis* and *Yarrowia lipolytica* [12]. Furthermore, it is a haploid and nonpathogenic yeast, with the associated advantages of genetic engineering and industrial safety [12]. Recently, whole-genome sequencing of *W. sorbophila* was completed, allowing easy access to the genetic information (https://www.ncbi.nlm.nih.gov/nuccore/NDIQ00000000.1/). Therefore, this emerging yeast is expected to greatly facilitate DCA production from fatty acid methyl esters, a vegetable oil-derived resource.

*Wickerhamiella sorbophila* was first isolated as a culture contaminant from washings of ion-exchange resins from a guanine monophosphate manufacturing plant [13]. Since its discovery, *W. sorbophila* has rarely been investigated, except for some short studies on its production of lactone [14] and chiral R-amino alcohol [15]. Here, we first described the development of effective genetic engineering tools for industrial application of *W. sorbophila*. To facilitate genetic engineering, selectable antibiotic-resistance markers were explored and used to remove target genes. To increase the probability of gene insertion into the target locus, the cell cycle of yeast was synchronized to the S/G2 phase, during which homologous recombination (HR) frequently occurs, for yeast transformation. Finally, we developed an engineered *W. sorbophila* strain with a blocked β-oxidation pathway and applied it to the biotransformation of DCA from methyl laurate.

## Results and discussion

### Cell growth in the presence of antibiotics

**Fig. 2:**
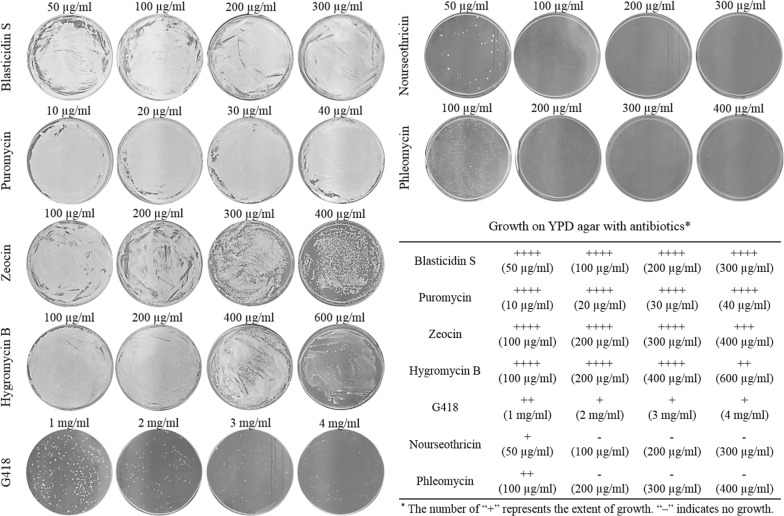
Antibiotic sensitivity of *W. sorbophila.* Cell growth was examined at various concentrations of antibiotics to identify a selectable marker. Cells (1 × 10^7^) were grown for 3 days on yeast peptone dextrose (YPD) agar medium containing blasticidin S, puromycin, Zeocin, hygromycin B, nourseothricin, or phleomycin. For comparison, cell growth is represented by “+” and “−” (+ ≤ 100 colonies; ++ ≤ 200 colonies; +++ ≤ 400 colonies; ++++ > 400 colonies)

Genetic manipulation is essential for the development of industrially competent strains. Despite the fact that the newly isolated *W. sorbophila* strain was a strong candidate for DCA production, biological information for genetic engineering of this strain is lacking. As a basic step for the development of genetic engineering tools, antibiotics for use of selectable markers in *W. sorbophila* were investigated. We evaluated cell growth in the presence of several antibiotics that are primarily used in eukaryotic genetic engineering (Fig. 2). No inhibitory effect on cell growth was observed with blasticidin S and puromycin. Zeocin, hygromycin B, and G418 exerted a slight inhibitory effect, but cell growth was still observed even at high concentrations. However, nourseothricin and phleomycin totally inhibited cell growth at 100 and 200 μg/ml, respectively. *Streptomyces noursei nat1* and *Streptoalloteichus hindustanus ble* genes reportedly confer resistance to nourseothricin and phleomycin, respectively [16, 17]. These genes were consequently used as selectable markers for *W. sorbophila*.

### Heterologous expression of *nat1* and *ble* genes

**Fig. 3:**
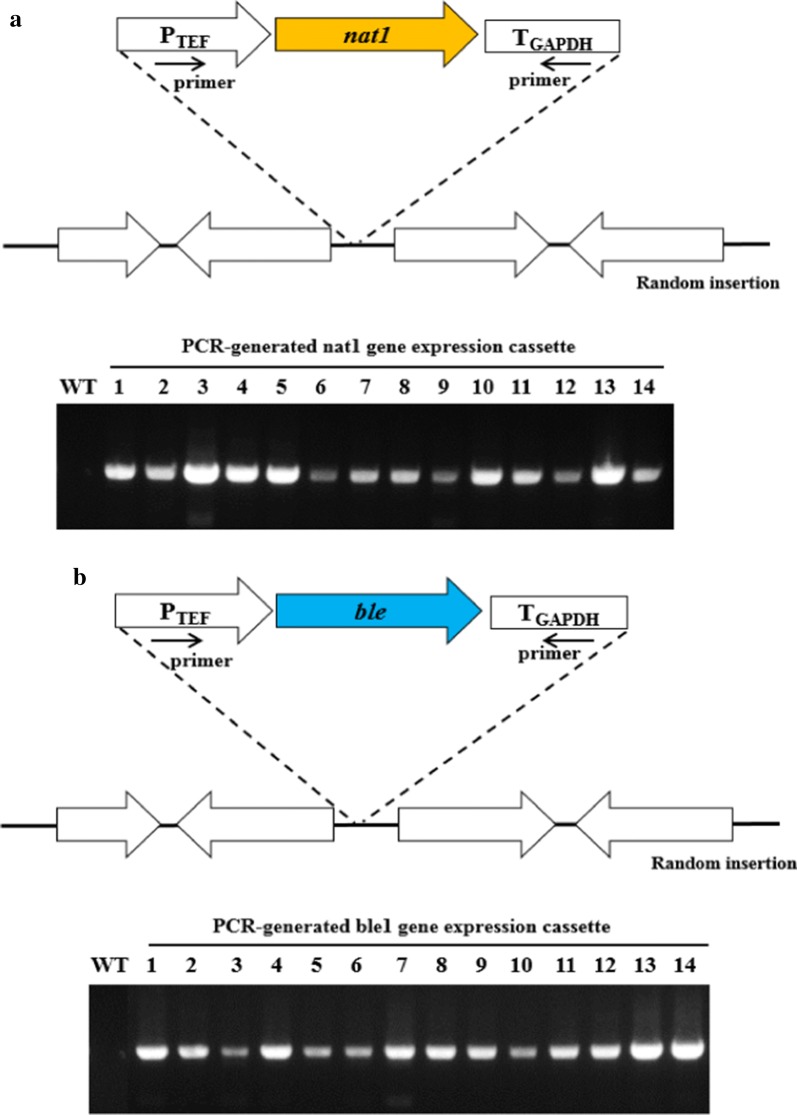
Schematic representation of the expression cassettes and chromosome integration in *W. sorbophila*. The pUC18-*nat1* (**a**) and pUC18-*ble* (**b**) cassettes, each containing the *TEF* promoter and *GAPDH* terminator, were PCR amplified using primers TEFp-F and GAPt-R and randomly inserted into the chromosome. The gel images show the results of yeast colony PCR using primers TEFp-F and GAPt-R. The sizes of the bands are 1.4 kb (**a**) and 1.2 kb (**b**)

Some yeasts, including the *Candida* species, decode CUG, a universal leucine codon, as serine. To enable heterologous expression of the marker genes, codon usage of *W. sorbophila* was analyzed. The analysis revealed that this strain might use standard genetic code (Additional file 1: Figure S1). Then, promoter and terminator sequences were investigated. While the use of heterologous promoter and terminator is often recommended to prevent interference caused by similarity of an endogenous DNA sequence during insertion of exogenous DNA into the chromosome, the stability or strength of expression is not guaranteed [18]. Hence, we next identified a homologous promoter sequence and a heterologous terminator sequence for heterologous gene expression. The 500-bp upstream region of the *TEF* gene was retrieved to identify a constitutive promoter in *W. sorbophila*. Translation elongation factor 1α gene, *TEF*, encoding the alpha subunit of the elongation factor complex, is involved in transferring the aminoacyl tRNA to the ribosome, and its promoter has been reported to constantly drive gene expression, regardless of the external environment and growth stage [19]. A heterologous terminator was obtained from the *C. tropicalis GAPDH* gene. To test the feasibility of using expression cassettes containing the *nat1* or *ble* gene, we transformed *W. sorbophila* with the constructs by allowing linearized DNA (the resistance cassettes) lacking homology arms to be randomly inserted into the chromosome (Fig. 3). After 4 days of incubation, nourseothricin- or phleomycin-resistant colonies appeared on the selection medium, and the inserted genes were identified by colony polymerase chain reaction (PCR) of the transformants. As shown in Fig. 3, the PCR products corresponding to the size of the inserted gene were detected in all resistant colonies tested, but not in the wild type. These observations indicated that the transcription system involving marker genes using a homologous promoter and heterologous terminator operated well in *W. sorbophila*.

### HR efficiency

**Fig. 4:**
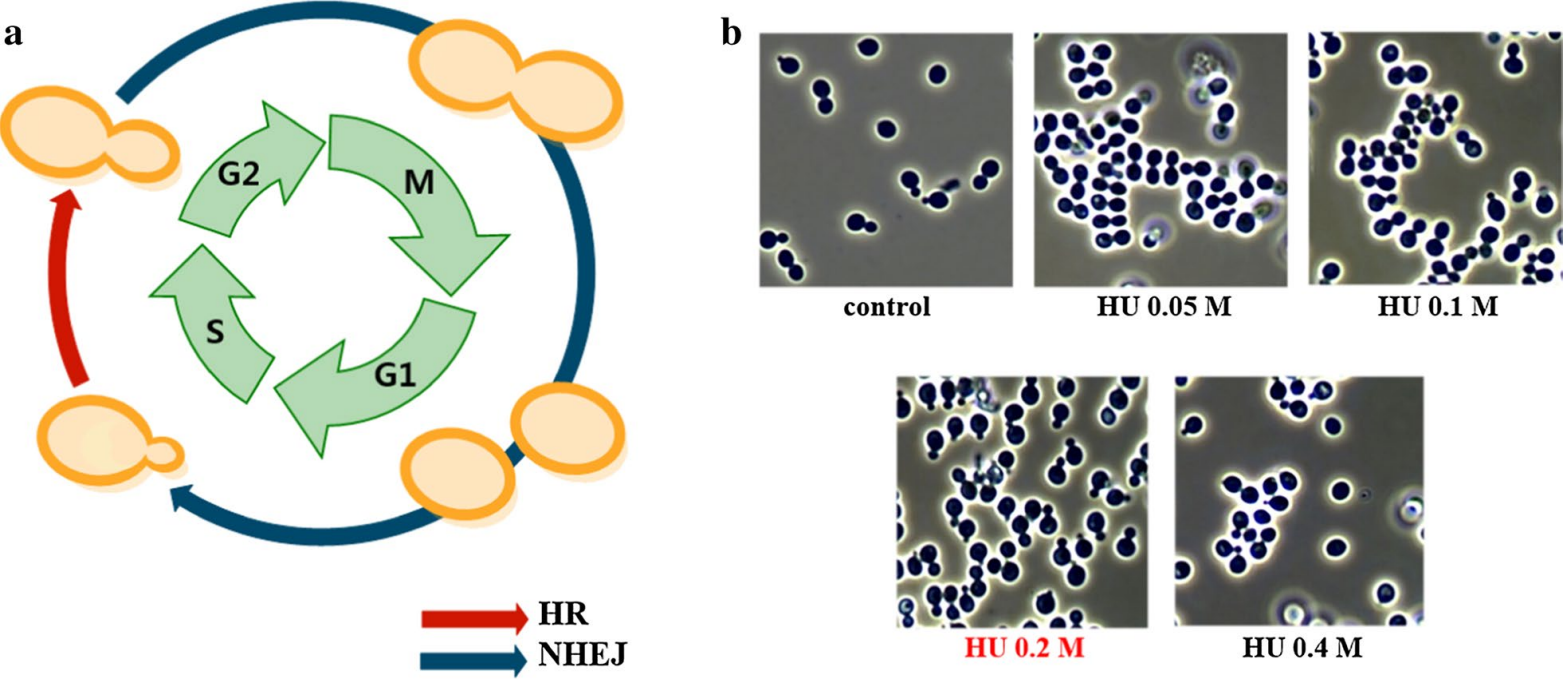
Cell synchronization using hydroxyurea (HU). In DNA repair, the HR and NHEJ mechanisms are competitive (**a**). HR is dominant at the S/G2 phase, and NHEJ is dominant at the G1/M phase. Micrographs of *W. sorbophila* treated with HU at 0, 0.05, 0.1, 0.2, and 0.4 M after 2 h (**b**)

In eukaryotes, double-strand breaks (DSBs) induce two kinds of DNA repair mechanisms: HR and non-homologous end joining (NHEJ). HR allows the truncated 3′-end strand to recognize and replicate intact homologous double strands. NHEJ directly connects two DSBs by annealing short sequences near their end [20]. These two mechanisms occur competitively, and the balance varies depending on the cell cycle or species [21]. In yeast genetic engineering, HR has been used for insertion of external DNA at a desired position in the chromosome. Nonconventional yeasts, such as *Y. lipolytica* and *Kluyveromyces marxianus*, reportedly predominantly use NHEJ, resulting in random integration. To increase the HR rate in nonconventional yeast, most studies have focused on eliminating the function of the genes associated with the NHEJ pathway, such as the ones encoding Ku70/80 [22, 23] and DNA ligase IV [24]. However, the errors of the highly conserved DNA repair system impair the maintenance of genome integrity. Mutations in the DNA repair system increase the susceptibility to DNA damage, and the resulting increased mutation rate reduces cell viability [25, 26]. To maintain an intact DNA repair system, we increased the HR rate by regulating the cell cycle. Recent studies have shown that the balance of HR and NHEJ shifts during the cell cycle [20, 21] (Fig. 4a). In yeast and mammalian cells, since the accessibility of sister chromatids—the preferred template for HR repair—affects HR efficiency, HR use is elevated between the S and G2 phases of the cell cycle, during which the sister chromatids can be used [27]. Therefore, we deleted the target genes by synchronizing the cells in the S phase using HU, an inhibitor of ribonucleotide reductase [28], and compared the HR efficiency in the resultant cells with that attained by other existing methods.

Prior to this experiment, the appropriate concentration of HU was determined. Microscopic analysis (Fig. 4b) revealed that cells at the S/G2/M phase coexisted at 0.05 and 0.1 M of HU, whereas cell division was inhibited at 0.4 M. Therefore, HU was used at 0.2 M to arrest the cells at the S/G2 phase. To compare the HR efficiency depending on the cell growth conditions, deletion cassettes containing selectable markers were constructed and integrated into the *URA3*, *ADE2*, *POX1*, and *POX2* loci, accordingly, depending on the growth stage (Additional file 1: Figure S2) (Table 2). *ura3* or *ade2* was eliminated using the *ble* marker; the resultant strains were named *W. sorbophila* U1 (***u****ra3Δ*) and *W. sorbophila* A1 (***a****de2Δ*). *pox1* or *pox2* was eliminated using the *URA3* marker; the resultant strains were named *W. sorbophila* UHP1 (***p****ox1Δ* with ***U****RA3* **h**arboring *glu*) and *W. sorbophila* UHP2 (***p****ox2Δ* with ***U****RA3* **h**arboring *glu*), respectively. In haploid yeast, the removal of genes with physiologically important functions is detrimental to cell growth and leads to a delay in the appearance of transformants [29]. To reduce the probability of selection errors, we supplemented the selection medium with the required nutrients, i.e., adenine and uracil, to support the growth of the desirable transformants. As shown in Table 2, HR frequency was highest in HU-treated cells, followed by those in the logarithmic and stationary phases of growth, in that order. HR efficiency at the *POX1* and *POX2* loci increased from 56.3% and 41.7%, respectively, to 97.9% for both cases. The original HR efficiency at the *URA3* and *ADE2* loci was nearly 0% in both early stationary and logarithmic phases, and increased to 4.8% and 25.6%, respectively, after HU treatment.

**Table 2:** HR efficiency depending on the growth stage

Growth stage	Strain	Target gene	Marker gene	HR efficiency
Early stationary phase	DS02	*ADE2*	*ble*	0% (1365)^a^
	DS02	*URA3*	*ble*	0% (186)^a^
	UHP1	*POX1*	*URA3*	56.3% (48)^a^
	UHP2	*POX2*	*URA3*	41.7% (48)^a^
Log phase	DS02	*ADE2*	*ble*	1% (1470)^a^
	DS02	*URA3*	*ble*	0% (186)^a^
	UHP1	*POX1*	*URA3*	81.3% (48)^a^
	UHP2	*POX2*	*URA3*	83.3 (48)^a^
S arrest	DS02	*ADE2*	*ble*	25.6% (379)^a^
	DS02	*URA3*	*ble*	4.8% (186)^a^
	UHP1	*POX1*	*URA3*	97.9% (48)^a^
	UHP2	*POX2*	*URA3*	97.9% (48)^a^

^a^Number of transformants screened

The experiment revealed some distinct features of *W. sorbophila*. The strain showed various HR efficiencies depending on the targeted gene locus. The HR efficiency of genes essential for growth, such as *ADE2* and *URA3*, was close to 0%, but that of *POX* genes involved in fatty acid metabolism exceeded 40% (Table 2). This suggested the occurrence of HR hotspots on the genome of *W. sorbophila*. We successfully removed the *ADE2* and *URA3* genes, typically characterized by nearly 0% HR efficiency, by regulating the cell cycle. Therefore, transformation involving HU is an effective and easy approach of increasing HR efficiency without permanently damaging the DNA repair system.

### Characterization of the putative *WsPOX* genes

Acyl-CoA oxidase (EC:1.3.3.6.), which catalyzes the initial step in fatty acid β-oxidation, transfers hydrogen atoms in the CH–CH group of acyl-CoA to molecular oxygen [30], resulting in the formation of trans-2-enoyl-CoA and H_2_O_2_. In alkane-assimilating yeasts, the deletion of *POX* genes prevents degradation of fatty acids, promoting ω-oxidation of the terminal methyl group [2]. There are six *POX* genes in *Y. lipolytica*, some of which exhibit specific substrate affinity, depending on the carbon chain length [31, 32]. Based on the whole-genome sequence of *W. sorbophila*, two putative *POX* genes with high amino acid sequence similarity to YALI0F10857g (the *Y. lipolytica POX*2 gene) were identified (*POX1*: B9G98_00908, 60% sequence similarity; *POX2*: B9G98_04160, 52% sequence similarity).

**Fig. 5:**
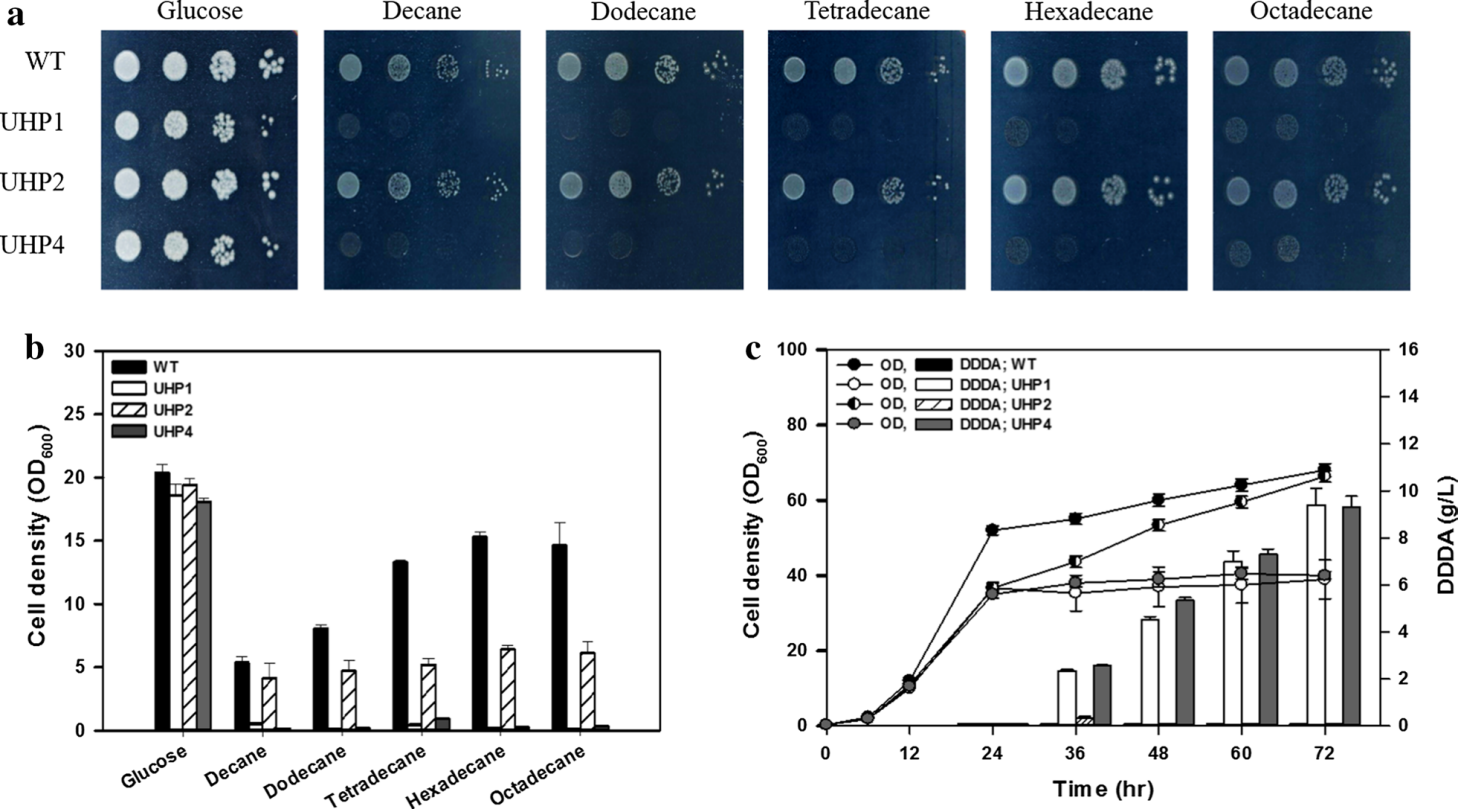
Characterization of *WsPOX*-deficient mutants. Growth of *POX*-deficient mutants on yeast nitrogen base (YNB) agar (**a**) and liquid (**b**) media containing various carbon sources (glucose, decane, dodecane, tetradecane, hexadecane, and octadecane). After 24-h growth, 1% (v/v) dodecane was added for DDDA conversion (**c**). Error bars represent the standard deviation of three independents trials. The values represent the means

We characterized the putative *POX* genes by analyzing *pox* gene-deficient mutants. The *pox1* and *pox2* deletion strains were obtained using the methods described above. For the sequential disruption of the *POX* genes, the *URA3* marker of *W. sorbophila* UHP1 was removed by HR between the both terminal *glu* sequences (pop-out) (*W. sorbophila* UHP3, ***p****ox1Δ* without ***U****RA3* **h**arboring *glu*), and then the *POX2* gene was eliminated by reusing the *URA3* marker (*W. sorbophila* UHP4, ***p****ox1Δ/pox2Δ* with ***U****RA3* **h**arboring *glu*). As shown in Fig. 5a, b, the overall growth of mutants was reduced in comparison with that of the wild-type strain. The *W. sorbophila* UHP2 strain grew in the presence of all tested carbon sources. However, the *W. sorbophila* UHP1 strain exhibited unaltered growth on glucose, but not in the presence of medium-chain (C_10_–C_12_) and long-chain (C_14_–C_18_) alkanes. Only the *POX1* gene—with a broad substrate spectrum—is expected to play an important role in fatty acid metabolism. An interesting phenomenon was reported previously, namely, that *W. sorbophila* simultaneously consumes various substrates during culture in the presence of mixed fatty acid methyl esters (C_10_–C_16_) [12]. This may be associated with the broad substrate specificity of the product of the *POX1* gene. The *POX2* gene, however, is considered to be a pseudogene or involved in the metabolism of short-chain fatty acids. The DDDA conversion test (Fig. 5c) revealed that only *W. sorbophila* UHP1 and *W. sorbophila* UHP4 were able to convert dodecane to DDDA. *W. sorbophila* UHP1 and *W. sorbophila* UHP4 produced 9.38 and 9.12 g/l of DDDA, respectively, and their conversion yields (mol/mol) were 90.1% and 92.6%, respectively. By contrast, the growth of *W. sorbophila* UHP2 was reduced during the first 24 h, similarly to the other mutants, but eventually metabolized and used dodecane for cell growth. Hence, removal of the *POX1* gene is essential for the accumulation of DDDA.

### Fed-batch fermentation for DDDA production

In *C. tropicalis*, a well-known DCA-producing yeast, the fermentation process for DCA production has already been optimized [2, 33, 34]. The fermentation for DCA production is divided into growth and conversion stages, where the medium pH greatly affects fatty acid and DCA solubility, DCA excretion and cell viability [11, 34]. During the conversion stage, acidic conditions (= pH 5.8) steadily promoted the cell growth, but DCA precipitates were formed and interfered with the process [35]. In the resting cells, a greater amount of DCA was released at a higher pH [36]; however, in living cells, pH values above 8.2 reduced cell viability and DCA productivity [34]. Considering this, we performed fed-batch fermentation using *W. sorbophila* UHP4 (with completely blocked β-oxidation) and methyl laurate. The pH was adjusted to 5.5 for the initial 12 h period to obtain high cell density, after which the pH was increased to 7.2–8.0 to solubilize fatty acid and DCA, and to promote DCA secretion (as described in detail in “Methods”).

**Fig. 6:**
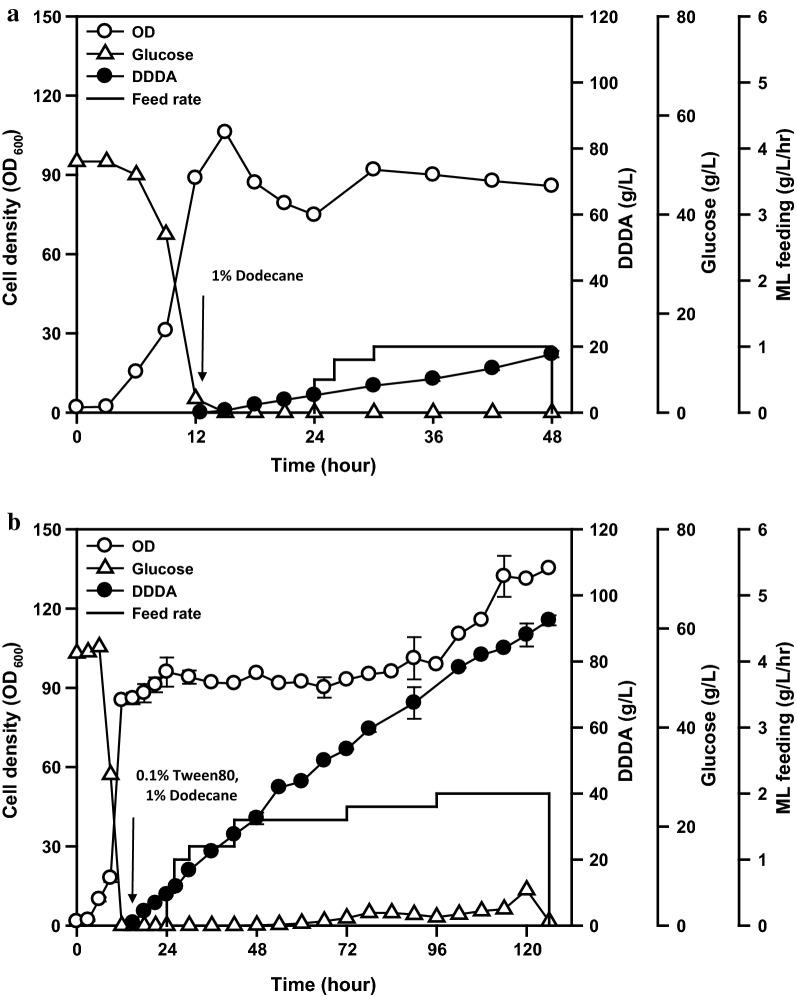
Time profile of DDDA production in the absence (**a**) and presence of Tween 80 (**b**) by *W. sorbophila* UHP4 in 5-l fed-batch fermentation. For DDDA production, dodecane was added to induce ω-oxidation-related enzymes, and methyl laurate was then fed continuously. *ML* methyl laurate. Error bars represent the standard deviation of three technical replicates. The values represent the means

DCA conversion is initiated by cytochrome P450 monooxygenase (CYP); members of the CYP family are alkane-inducible in alkane-assimilating yeasts [37–39]. In *C. tropicalis*, the addition of methyl decanoate without the pre-activation of ω-oxidation resulted in the accumulation of decanoic acid and directly affected cell viability [11]. Thus, prior to methyl laurate addition, 1% (v/v) dodecane of the culture was added to pre-activate the ω-oxidation pathway of *W. sorbophila*. In addition, Tween 80, a nonionic surfactant esterified with polyethoxylated sorbitan and oleic acid, was used to facilitate the uptake of dodecane. In various prokaryotic and eukaryotic microorganisms, cellular uptake of hydrophobic substrates is involved in several mechanisms, such as substrate solubilization by extracellular emulsifying agents, modification of the cell surface to facilitate adhesion [40], passive diffusion via the cell membrane [33], and transporter-mediated uptake [41]. In *C. tropicalis*, the alkane-binding affinity of glucose-grown cells was lower than that of alkane-grown cells due to the alteration of the cell wall components [42]; the uptake of alkane on glucose-grown cells showed a greater dependence on the emulsification of alkane than alkane-grown cells [42]. Based on these reports, 1% (v/v) Tween 80 was added to dissolve dodecane in the culture medium. Even if Tween 80 was hydrolyzed, as observed in some yeasts [43], oleic acid resulting from Tween 80 cleavage is not expected to affect the amount of DDDA produced by the strain with blocked β-oxidation. Finally, *W. sorbophila* UHP4 produced 92.5 g/l of DDDA over 126 h, with the productivity and yield of 0.83 g/l/h and 0.86 (mol/mol), respectively (Fig. 6b). This was 1.4 times higher than the fermentation data for *C. tropicalis* and methyl laurate (Table 1). Most studies with dodecane have reported higher values than those obtained in the current study; however, an accurate comparison was difficult because the DDDA concentration was determined by pH titration or calculated based on the initial volume (Table 1). In the absence of Tween 80, DDDA productivity was greatly reduced, almost by half (Fig. 6a). A detailed study of this phenomenon would aid the improvement of the *W. sorbophila* performance. Furthermore, alongside process optimization, future studies on biotransformation using fatty acid methyl esters with various carbon chain lengths would expand the scope for the application of this strain.

## Conclusions

Currently, sustainable production of DCA is a major concern in industrial biotechnology. Although many studies have contributed to this field, the biotransformation of DCA from renewable resources has been hindered because of the cellular toxicity of fatty acids. Previously, we identified several positive aspects of DCA production—industrially and biologically—using a *W. sorbophila* strain [12]. Here, we developed a novel microbial platform for DCA production, overcoming the limitation of toxicity. Based on whole-genome sequence information for *W. sorbophila*, we developed and optimized genetic engineering tools. To increase HR efficiency during the yeast transformation process, the cell cycle was artificially fixed at the S/G2 phase. To prevent the metabolism of alkanes and fatty acids, putative *POX* genes were characterized and removed. Interestingly, only *pox1*-deficient strains produced DDDA, suggesting that the *POX1* gene plays a major role in β-oxidation. In 5-l fed-batch fermentation with *W. sorbophila* UHP4, 92.5 g/l of DDDA was produced from methyl laurate in 126 h. In conclusion, the presented data demonstrated that *W. sorbophila* is a promising microbial platform for DCA production from vegetable oil-based resources. Furthermore, the developed genetic engineering tools will form the basis for future research for the production of various value-added oleochemicals derived from vegetable oil, together with traditional metabolic engineering strategies, to improve DCA productivity.

## Methods

### Strains, materials, and growth conditions

The strains used in the current study are listed in Table 3. The *nat1* and *ble* genes were synthesized by GenScript (Piscataway, NJ, USA) (Additional file 1: Table S1). YNB medium without amino acids, and yeast extract and peptone were purchased from Becton-Dickinson (Franklin Lakes, NJ, USA). Nourseothricin was purchased from BioVision (Milpitas, CA, USA). Blasticidin S, hygromycin B, phleomycin, puromycin, and Zeocin were purchased from InvivoGen (San Diego, CA, USA). Adenine, uracil, HU, alkanes, lauric acid, methyl laurate, DDDA, Tween 80, and *N*,*O*-bis-trimethylsilyl-trifluoroacetamide were from Sigma-Aldrich (St. Louis, MO, USA). 5-Fluoroorotic acid (5-FOA) and tetradecanedioic acid were from TCI Chemical (Tokyo, Japan). Antifoam agent (adekanol LG-109) was from Adeka (Tokyo, Japan). All other chemicals were purchased from Junsei Chemical (Tokyo, Japan).

**Table 3:** Strains used in the current study

Strain	Genotype	Reference
*W. sorbophila* DS02	Wild type	[12]
*W. sorbophila* U1	*ura3Δ::Sable*	This work
*W. sorbophila* A1	*ade2Δ::Sable*	This work
*W. sorbophila* UHP1	*ura3Δ pox1Δ::glu*-*CtURA3*-*glu*	This work
*W. sorbophila* UHP2	*ura3Δ pox2Δ::glu*-*CtURA3*-*glu*	This work
*W. sorbophila* UHP3	*ura3Δ pox1Δ::glu*	This work
*W. sorbophila* UHP4	*ura3Δ pox1Δ::glu pox2Δ::CtURA3*	This work

The yeast strains were routinely cultured at 30 °C in YPD medium. When required, the medium was supplemented with 100 mg/l adenine or 100 mg/l uracil. For the selection of antibiotic markers, 1 × 10^7^ cells were spread on YPD agar medium containing 50–300 μg/ml blasticidin S, 10–40 μg/ml puromycin, 100–400 μg/ml Zeocin, 100–600 μg/ml hygromycin B, 1–4 mg/ml G418, 50–300 μg/ml nourseothricin, or 100–400 μg/ml phleomycin. Antibiotic-resistant colonies were observed after incubation at 30 °C for 3 days.

To analyze the growth of *W. sorbophila* strains, appropriate carbon sources [20 g/l glucose, 1% (v/v) decane, 1% (v/v) dodecane, 1% (v/v) tetradecane, 1% (v/v) hexadecane, or 1% (v/v) octadecane] were added to YNB medium with 0.1% (v/v) Tween 80. In the case of solid YNB medium, alkanes were supplied in the vapor phase, as described previously [44]. Alkanes with fewer than 8 or over 20 carbons were excluded because of their toxicity, volatility, or melting temperature. Pre-cultured cells were washed with YNB medium and then spotted onto agar medium in tenfold serial dilutions starting with 10 μl of suspensions with OD_600_ of 0.1.

### Prediction of codon usage

The CUG codon usage of *W. sorbophila* was predicted using the fungal CUG codon-predicting site Bagheera [45]. The whole-genome sequence information was obtained from the GenBank database (Accession Number NDIQ00000001.1).

### PCR amplification

The *nat1* and *ble* genes were PCR amplified using primers nat1-F and nat1-R, and ble-F and ble-R, respectively. A 500-bp sequence upstream of the *TEF* gene was amplified for use as a promoter from the genomic DNA of *W. sorbophila* DS02 using primers TEFp-F and TEFp-R. The 500-bp 5′ and 3′ homologous arms of the *ADE2*, *URA3*, *POX1*, and *POX2* genes were amplified for target gene disruption from the genomic DNA of *W. sorbophila* DS02 using primers ADE2 HR-F1, ADE2 HR-R2, ADE2 HR-F3, and ADE2 HR0-R4, for *ADE2* disruption; URA3 HR-F1, URA3 HR-R2, URA3 HR-F3, and URA3 HR-R4, for *URA3* disruption; POX1 infu-F1, POX1 infu-R2, POX1 infu-F3, and POX1 infu-R4, for *POX1* disruption; and POX2 infu-F1, POX2 infu-R2, POX2 infu-F3, and POX2 infu-R4, for *POX2* disruption. The genomic sequence information was obtained from the GenBank database (Accession Number NDIQ00000000.1). A 300-bp fragment downstream of the *GAPDH* gene sequence was amplified for use as a terminator from the genomic DNA of strain ATCC 20962 using primers CtGAPt-F and CtGAPt-R; the genomic sequence information was obtained from the GenBank database (Accession Number HQ171163.1). A 1537-bp fragment of the *glu* gene was amplified for use as both flanking sequence of the *URA3* marker from the genomic DNA of *Bacillus subtilis* using primers glu-F and glu-R. The genomic sequence information for that strain was obtained from the GenBank database (Accession Number CP020102.1).

Yeast colony PCR was performed to select transformants. Single colonies grown on the selection medium were suspended in 30 μl of distilled water, treated with 20 U of lyticase, and incubated for 1 h at 37 °C. The resultant solution was directly used as a source of template for PCR. The insertion of selectable markers into transformants was confirmed by colony PCR using primers CiTEFp-F and CtGAPt-R. Disruption of target genes was first confirmed by colony PCR, and then by PCR with the genomic DNA isolated from the transformants, using primers URA3con-F, URA3con-R, POX1con-F, POX2con-F, and POXcon-R, as appropriate, specific for the sequence outside of the homologous arms. All primers used in the current study are listed in Additional file 1: Table S2.

### Plasmid construction

To construct a heterologous gene expression cassette, the *TEF* promoter, *nat1* gene, and *GAPDH* terminator were inserted into the *Apa*I/*Xho*I sites of pUC18 vector. The resulting plasmid was termed pUC18-*nat1*. The *nat1* gene in the pUC18-*nat1* vector was then replaced by the *ble* gene, which resulted in plasmid pUC18-*ble*.

The 5′ and 3′ homologous arms of the *ADE2* gene were inserted into the *Mlu*I/*Apa*I and *Bgl*II/*Eco*RI sites of the pUC18-*ble* vector, respectively. The resulting plasmid was termed pUC18-*ADE2*-*ble*. Homologous arms of the *URA3* gene were inserted in the same way, resulting in a plasmid termed pUC18-*URA3*-*ble*.

For sequential disruption of the *W. sorbophila POX* genes, *URA*3 pop-out cassette was constructed according to a previously published protocol [46]. The *W. sorbophila URA3* fragment was inserted into the *Bam*HI/*Bgl*II sites of pGEM-T easy vector, and the *glu* gene was inserted into the *Bam*HI/*Bgl*II sites at both ends of *URA3*, as a repeating sequence in the vector. The resulting plasmid contained the *glu*-*URA3*-*glu* cassette and was termed pGEM-*gug*. Then, the 5′ and 3′ homologous arms of the *POX1* gene were inserted into the *Apa*I/*Sph*I and *Mlu*I sites, respectively, of pGEM-*gug* vector, which was termed pGEM-*POX1*-*gug*. The homologous arms of the *POX2* gene were inserted into the *Apa*I/*Sph*I and *Nde*I/*Sal*I sites, respectively, of pGEM-*gug* vector, resulting in the plasmid pGEM-*POX2*-*gug.*

### Transformation

*Wickerhamiella sorbophila* was transformed using the lithium acetate method, with some modification [47]. After transformation of cells with 1 μg of the linearized cassette, the cell pellet was suspended in 1 ml of YPD and incubated for 2 h at 30 °C with shaking at 180 rpm. When required, the medium was supplemented with 100 mg/l adenine or 100 mg/l uracil. Then, the cells were spread on the selection medium and incubated for 5 days at 30 °C. *Ura3*-deletion candidates were confirmed by colony PCR after primary counter-selection on 5-FOA agar medium [48]; *ade2*-deletion candidates were simply identified by red/white colony color [49]. Putative *pox* gene-deletion candidates were confirmed by colony PCR.

### Cell synchronization using HU

Synchronization of *W. sorbophila* cells was performed according to a previously published protocol [50]. Subsequently, subcultured cells were grown to OD_600_ = 1 in 50 ml of YPD medium (when required, 100 mg/l adenine or 100 mg/l uracil was included in the medium), treated with HU, and incubated for 2 h at 30 °C with shaking at 180 rpm. The synchronized cells were used for yeast transformation.

### Marker recycling

To remove the *URA3* marker from the *W. sorbophila* UHP1 genome, the cells were grown in 3 ml of YPD medium overnight. Further, 100 μl of the culture was spread on 5-FOA agar medium and incubated for 5 days at 30 °C. The identity of the obtained colonies was confirmed by colony PCR.

### Flask cultivation for DCA production

One loopful of cells was pre-cultured in a 15-ml round tube with 3 ml of YPD medium overnight. Then, 1 ml of the culture was transferred to a 250-ml baffled flask containing 20 ml of YPDP medium (10 g/l yeast extract, 20 g/l peptone, 30 g/l glucose, and 1.8 g/l KH_2_PO_4_) and incubated at 30 °C with shaking at 200 rpm. After culturing for 24 h, 0.3 g (15 g/l) K_2_HPO_4_ and 0.2 ml (1%, v/v) of dodecane were added to the culture broth; 0.1 g (5 g/l) glucose was added, as 500 g/l glucose solution, after 24, 30, 36, 48, and 60 h.

### Fed-batch fermentation

Fed-batch fermentation using the engineered *W. sorbophila* strain was performed according to a previously described protocol, with some modification [12]. One loopful of cells grown on YPD agar plates was inoculated in a 250-ml baffled flask containing 20 ml of YPD medium and incubated at 30 °C with shaking at 200 rpm for 24 h. The culture was transferred to a 2-l baffled flask containing 200 ml of YPD medium and incubated at 30 °C and 200 rpm for 24 h. Subcultured cells were transferred to a 5-l fermenter (CNS, Daejeon, Korea) with 2 l of medium containing 50 g/l glucose, 20 g/l yeast extract, 8 g/l (NH_4_)_2_SO_4_, 2 g/l K_2_HPO_4_, 0.1 g/l NaCl, 0.1 g/l CaCl_2_·H_2_O, and 0.5 ml of antifoam agent. Temperature, medium pH, and aeration were controlled at 30 °C, 5.5, and 1 vvm, respectively. Dissolved oxygen was maintained above 30% by modulating the agitation speed between 200 and 900 rpm. After culturing for 12 h, 20 ml (1%, v/v) of dodecane and 2 ml (0.1%, v/v) of Tween 80 were added to the culture broth; glucose was continuously added at a rate of 2 g/l/h; the medium pH was adjusted from 5.5 to 7.2 with 8 N NaOH, and then increased up to 7.8 (pH 7.2 for the culture period of 12–24 h; pH 7.4 for 24–48 h; pH 7.6 for 48–96 h; pH 7.8 for 96–126 h; and pH 8.0 for 120–126 h). After 24 h, methyl laurate was continuously added at a rate of 0.5–2 ml/l/h (0.5 ml/l/h for the culture period of 24–26 h; 1 ml/l/h for 26–30 h; 1.2 ml/l/h for 30–42 h; 1.6 ml/l/h for 42–72 h; 1.8 ml/l/h for 72–96 h; and 2 ml/l/h for 96–126 h) (Fig. 6b). The feeding rate was calculated based on the initial volume, and product concentration and productivity were calculated based on the end volume.

### Analytical methods

Cell density, the concentrations of glucose, and DDDA in the culture broth were determined. Cell density was determined using an ultraviolet spectrophotometer (Uvikon XL; Secomam, Alès, France) by measuring the absorbance at 600 nm. Glucose concentration was determined using a glucose analyzer (YSI 2700; YSI Life Sciences, Yellow Springs, OH, USA). DDDA was analyzed by gas chromatograph (Master GC; Dani Instruments, Cologno Monzese, Italy) equipped with an RTX-5 column (Restek, Bellefonte, PA, USA). A 50-μl aliquot of 6 N H_2_SO_4_ was added to 100 μl of culture broth, with tetradecanedioic acid used as an internal standard. The sample was then mixed with 400 μl of diethyl ether. The upper solvent was separated and silylated with *N*,*O*-bis(trimethylsilyl)trifluoroacetamide. Each measurement was performed three times.

## Additional file

**Additional file 1: Table S1.** The sequences of *nat1* and *ble* genes used in the current study. **Table S2.** Primers used in the current study. **Figure S1.** CUG codon prediction for *Wickerhamiella sorbophila.* The genome sequence uploaded in Bagheera was matched with sequences of 2071 proteins from 38 different protein families using TBALSTN [1]. **Figure S2.** Schematic representation of the deletion cassettes and chromosome integration in *W. sorbophila*. The pUC18-*URA3-ble* cassette was PCR-amplified using primers URA3-F and URA3-R, and inserted into the *URA3* locus (a). The pUC18-*ADE2-ble* cassette was PCR-amplified using primers ADE2 HR-F and ADE2 HR-R, and inserted into the *ADE2* locus (b). The pGEM-*POX1*-*gug* cassette was linearized by *Hpa*I digestion and inserted into the *POX1* locus (c). The pGEM-*POX2*-*gug* cassette was linearized by *Sma*I digestion and inserted into the *POX2* locus (d).

## Abbreviations

5-FOA: 5-fluoroorotic acid
CYP: cytochrome P450 monooxygenase
DCA: dicarboxylic acid
DDDA: dodecanedioic acid
DSB: double-strand break
GC: gas chromatography
HR: homologous recombination
HU: hydroxyurea
NHEJ: non-homologous end joining
PA: polyamide
PCR: polymerase chain reaction
YNB: yeast nitrogen base
YPD: yeast peptone dextrose
