# Supplementary material for: Development of a promising microbial platform for the production of dicarboxylic acids from biorenewable resources
Source: Biotechnol Biofuels. 2018 Nov 9;11:310. doi: 10.1186/s13068-018-1310-x (PMC6225622; doi:10.1186/s13068-018-1310-x)
Supplement: Supplementary file 1 — Additional file 1: Table S1. The sequences of nat1 and ble genes used in the current study. Table S2. Primers used in the current study. Figure S1. CUG codon prediction for Wickerhamiella sorbophila. The genome sequence uploaded in Bagheera was matched with sequences of 2071 proteins from 38 different protein families using TBALSTN [1]. Figure S2. Schematic representation of the deletion cassettes and chromosome integration in W. sorbophila. The pUC18-URA3-ble cassette was PCR-amplified using primers URA3-F and URA3-R, and inserted into the URA3 locus (a). The pUC18-ADE2-ble cassette was PCR-amplified using primers ADE2 HR-F and ADE2 HR-R, and inserted into the ADE2 locus (b). The pGEM-POX1-gug cassette was linearized by HpaI digestion and inserted into the POX1 locus (c). The pGEM-POX2-gug cassette was linearized by SmaI digestion and inserted into the POX2 locus (d). [file 13068_2018_1310_MOESM1_ESM.docx]

**Additional File 1**

**Development of a promising microbial platform for the production of dicarboxylic acids from biorenewable resources**

Heeseok Lee^1,2^, Changpyo Han^1^, Hyeok-Won Lee^1^, Gyuyeon Park^1,2^, Wooyoung Jeon^1^, Jungoh Ahn^1^, Hongweon Lee^1^

^1^Biotechnology Process Engineering Center, Korean Research Institute of Bioscience and Biotechnology (KRIBB), 30 Yeongudanji-ro, Cheongwon-gu, Cheongju-si, Chungcheongbuk-do 28116, Republic of Korea

^2^Department of Bioprocess Engineering, KRIBB School of Biotechnology, Korea University of Science and Technology (UST), 217 Gajeong-ro, Yuseong-gu, Daejeon 34113, Republic of Korea

*Corresponding author: Hongweon Lee, E-mail: hwlee@kribb.re.kr, Tel.: +82-43-240-6612, Fax: +82-43-240-6609

Co-authors:

Heeseok Lee (lhs88@kribb.re.kr)

Changpyo Han (cphan525@kribb.re.kr)

Hyeok-Won Lee (tntn7616@kribb.re.kr)

Gyuyeon Park (gyuyeon@kribb.re.kr)

Wooyoung Jeon (wyjeon27@kribb.re.kr)

Jungoh Ahn (ahnjo@kribb.re.kr)

**Table S1.** The sequences of *nat1* and *ble* genes used in the current study

| **Gene** | **Sequence** |
| --- | --- |
| *nat1* | ATGACCACTTTGGATGACACCGCTTACAGATACAGAACAAGCGTTCCAGGTGATGCTGAAGCTATTGAAGCATTGGACGGTAGTTTTACTACTGATACTGTTTTTAGAGTTACCGCCACCGGTGATGGATTCACTTTGAGAGAAGTCCCTGTTGATCCACCACTTACCAAGGTTTTCCCAGACGACGAATCTGACGATGAGTCCGACGACGGTGAGGATGGTGATCCAGACTCCAGAACTTTTGTCGCTTATGGGGACGATGGCGACTTAGCCGGTTTCGTGGTTATTTCGTATTCTGCCTGGAACCGTAGATTAACCGTTGAAGATATCGAAGTCGCTCCTGAACATAGAGGCCACGGTGTCGGTAGAGCTTTGATGGGTTTGGCTACTGAATTTGCCGGAGAGAGAGGTGCTGGCCACTTGTGGTTGGAAGTGACGAATGTCAACGCTCCAGCTATCCATGCATATAGAAGGATGGGTTTCACTTTATGTGGTTTGGATACAGCCTTGTACGACGGTACCGCTTCCGATGGTGAAAGACAAGCCTTGTACATGTCAATGCCATGTCCATGA |
| *ble* | ATGTCGAAGTTGACTTCTGCAGTCCCAGTTTTAACCGCTCGTGACGTCGCTGGCGCCGTTGAATTTTGGACTGATAGATTGGGATTCTCAAGAGATTTCGTTGAAGATGACTTTGCTGGTGTTGTTAGAGATGACGTTACCTTGTTCATCTCCGCCGTCCAAGACCAGGTTGTGCCAGACAACACATTGGCCTGGGTTTGGGTCAGAGGTCTTGATGAGTTGTACGCTGAATGGAGTGAAGTTGTGTCTACCAACTTTAGAGATGCCTCCGGTCCAGCAATGACTGAAATTGGTGAACAACCTTGGGGTAGAGAATTTGCTTTAAGAGATCCAGCTGGTAATTGTGTCCACTTCGTCGCTGAGGAACAAGACTGA |

**Table S2.** Primers used in the current study

| **Name** | **Sequence (5′ → 3′)** | **Restriction sites** |
| --- | --- | --- |
| TEFp-F | CgggcccAAGATATATAATAGCCACCTCACCCC | *Apa*I |
| TEFp-R | TgagctcGCGGTTAGCAAATTGTTCAAC | *Sac*I |
| CtGAPt-F | AaagcttCTATCCAACAAACTCTAGGGGT | HindIII |
| CtGAPt-R | TctcgagTCTGGTTTAGAAGTAGGGACTGTAT | *Xho*I |
| nat1-F | acgcgtgggcccctcgagATGACCACTTTGGATGACAC | *Mlu*I*, Apa*I *Sac*I |
| nat1-R | ctcgagaagcttTTATCATGGACATGGCATTGACA | *Hind*III, *Xho*I |
| ble-F | acgcgtgggcccctcgagATGTCGAAGTTGACTTCTGC | *Mlu*I*, Apa*I *Sac*I |
| ble-R | agatctctcgagaagcttTCAGTCTTGTTCCTCAGCG | *Hind*III, *Xho*I, *Bgl*II |
| ADE2 HR –F1 | ACGAAGTTATacgcgtACTCGATTGCATTACATTAGAAAA | *Mlu*I |
| ADE2 HR –R2 | CTATTATATATCTTgggcccATCATTGCTGGTGCTGGT | *Apa*I |
| ADE2 HR –F3 | ACGAACGGTAagatctTCAATTTCAATTGTGACGACA | *Bgl*II |
| ADE2 HR –R4 | CCATGATTACgaattcTCCGCCGAAATATAATATAAAGTT | *EcoR*I |
| ADE2 HR-F | ACTCGATTGCATTACATTAGAAAAATAG |  |
| ADE2 HR-R | TCCGCCGAAATATAATATAAAGTTTG |  |
| URA3 HR –F1 | ACGAAGTTATacgcgtTTTTCAGGCCATGAAAAAGCAG | *Mlu*I |
| URA3 HR –R2 | CTATTATATATCTTgggcccCAGCAGTACCGCCC | *Apa*I |
| URA3 HR –F3 | CTAAACCAGActcgagATGTGCGTTTTAACCATGC | Xho*I* |
| URA3 HR–R4 | CCATGATTACgaattcTCTTGTTCGCAAAAATCTCG | *EcoR*I |
| URA3-F | TTTTCAGGCCATGAAAAAGCAG |  |
| URA3-R | CTCTTGTTCGCAAAAATCTCG |  |
| URA3con-F | CCATAATCGGGGATTCCAAG |  |
| URA3con-R | AAGACCTCTGACCCAAACCCAGGCCAATGTGTTG |  |
| glu-F | TggatccACGAAGCTTTAGCAATCGC | *BamH*I |
| glu-R | TagatctCCTACATCACCGAGAACGAC | *Bgl*II |
| CiURA3-F | ggatccGCTGGTTGTATGGAGGCG | *Hpa*I |
| CiURA3-R | agatctCATGGAACGAACGCGC |  |
| POX1 infu-F1 | TTGGGCCCGACGTCGgttaacCCGGTCTCTACCGCGTA | *Hpa*I |
| POX1 infu-R2 | CATGGCGGCCGGGAGGACGTCGTTAGCGGGG |  |
| POX1 infu-F3 | AGAGCTCCCAACGCGTCGTCTTTCGACAGAATTATATATTG |  |
| POX1 infu-R4 | TATGCATCCAACGCGgttaacAGTACCCTTTGTCTGCGTTG | *Hpa*I |
| POX2 infu-F1 | TTGGGCCCGACGTCGcccgggGCGTCACCAACAGTTTCC | *Sma*I |
| POX2 infu-R2 | CATGGCGGCCGGGAGGAAAACGAAGTGAGGGAGG |  |
| POX2 infu-F3 | CCGCCTGCAGGTCGAATTTGTTTTTTGTGCGAATGT |  |
| POX2 infu-R4 | GGGAGCTCTCCCATAcccgggCAGGCGTGCCTCTAAACTAG | *Sma*I |
| POX1con-F | AACCCCCGTCACGTGG |  |
| POX2con-F | CTAGCAGTGCCGTTTGAAGG |  |
| POXcon-R | GGAGCTTTCCTCCAAAGGC |  |


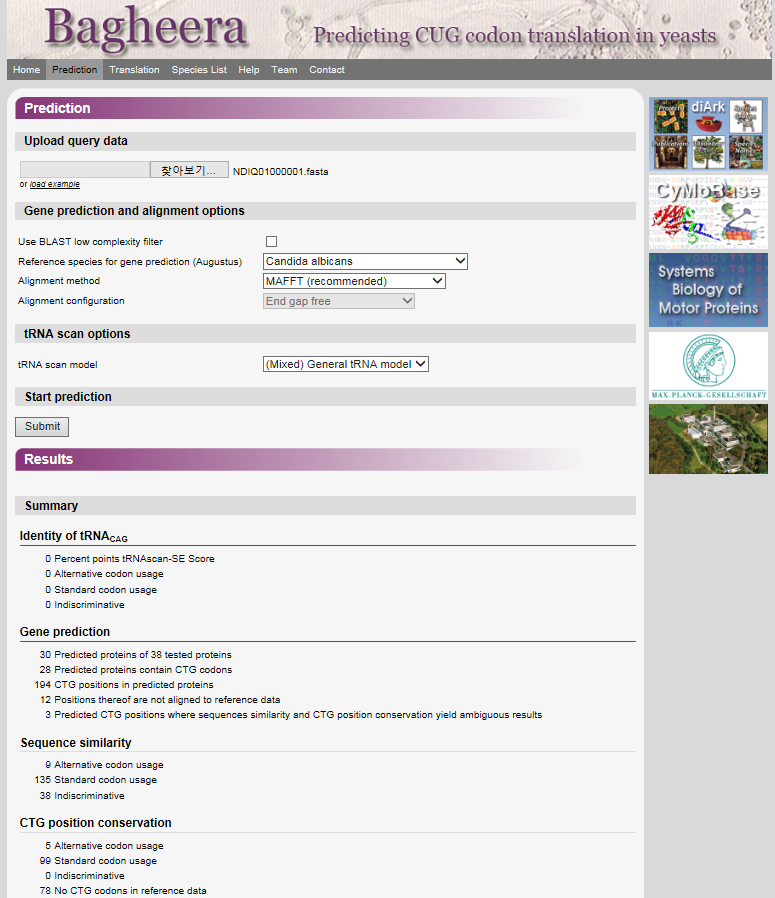


**Figure S1.** CUG codon prediction for *Wickerhamiella sorbophila.* The genome sequence uploaded in Bagheera was matched with sequences of 2071 proteins from 38 different protein families using TBALSTN [[1](#_ENREF_1)].


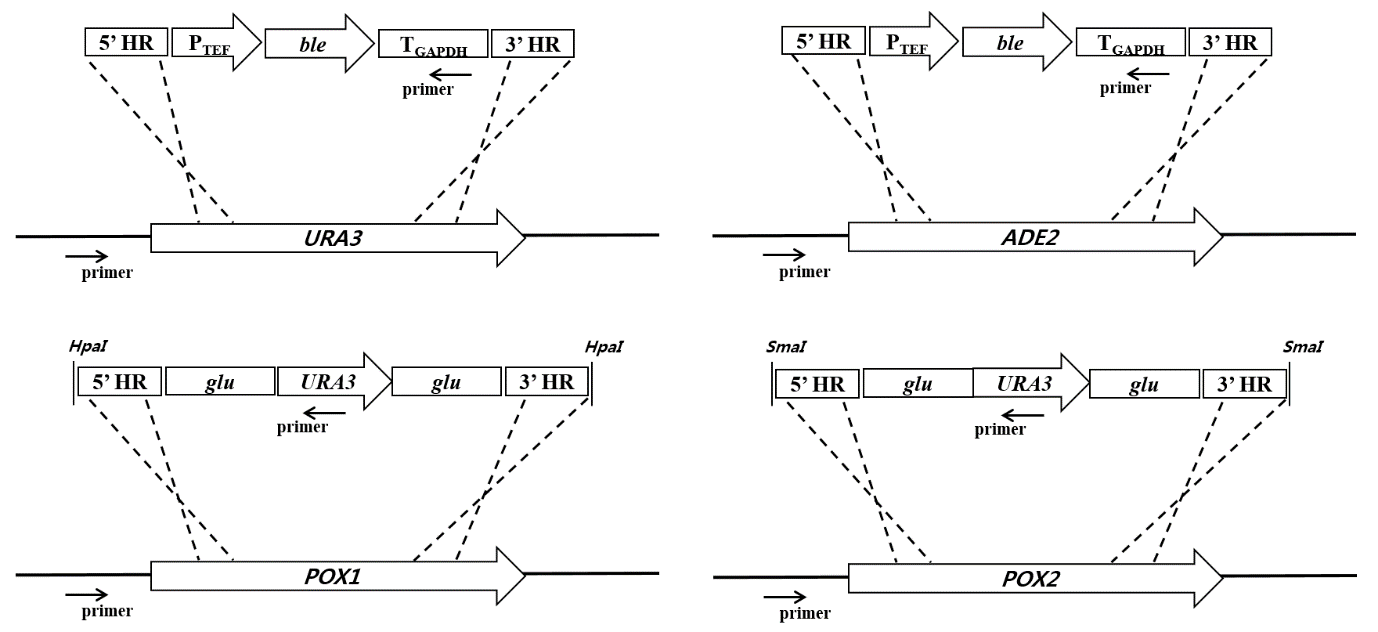


(c)

(d)

(a)

(b)

**Figure S2.** Schematic representation of the deletion cassettes and chromosome integration in *W. sorbophila*. The pUC18-*URA3-ble* cassette was PCR-amplified using primers URA3-F and URA3-R, and inserted into the *URA3* locus (a). The pUC18-*ADE2-ble* cassette was PCR-amplified using primers ADE2 HR-F and ADE2 HR-R, and inserted into the *ADE2* locus (b). The pGEM-*POX1*-*gug* cassette was linearized by *Hpa*I digestion and inserted into the *POX1* locus (c). The pGEM-*POX2*-*gug* cassette was linearized by *Sma*I digestion and inserted into the *POX2* locus (d).

**References**

1. Mühlhausen S, Kollmar M: **Predicting the fungal CUG codon translation with Bagheera**. *BMC genomics* 2014, **15**(1):411.
